# High prevalence of peripheral neuropathy in multiple myeloma patients and the impact of vitamin D levels, a cross-sectional study

**DOI:** 10.1007/s00520-021-06414-3

**Published:** 2021-07-17

**Authors:** B. E. Oortgiesen, J. A. Kroes, P. Scholtens, J. Hoogland, P. Dannenberg - de Keijzer, C. Siemes, F. G. A. Jansman, R. E. Kibbelaar, N. J. G. M. Veeger, M. Hoogendoorn, E. N. van Roon

**Affiliations:** 1 Department of Clinical Pharmacy and Pharmacology, Medical Centre Leeuwarden, Leeuwarden, The Netherlands; 2 Unit of Pharmacotherapy, Epidemiology and Economics, Department of Pharmacy, University of Groningen, Groningen, The Netherlands; 3 Department of Haematology, Deventer Hospital, Deventer, The Netherlands; 4 Department of Clinical Pharmacy and Pharmacology, Deventer Hospital, Deventer, The Netherlands; 5 Department of Pathology, Pathology Friesland, Leeuwarden, The Netherlands; 6 Department of Epidemiology, MCL Academy, Leeuwarden, The Netherlands; 7 Department of Epidemiology, University of Groningen, University Medical Centre Groningen, Groningen, The Netherlands; 8 Department of Haematology, Medical Centre Leeuwarden, Leeuwarden, The Netherlands

**Keywords:** Multiple myeloma, Peripheral nervous system diseases, Vitamin D, Drug therapy, Multi-centre cohort study

## Abstract

**Purpose:**

Peripheral neuropathy (PN) is common in patients with multiple myeloma (MM). We hypothesized that the relationship between hypovitaminosis D and PN described in diabetes mellitus patients may also be present in MM patients.

**Methods:**

To study this potential association, we assessed the incidence of hypovitaminosis D (vitamin D < 75 nmol/L [= 30 ng/mL]) in smouldering and active MM patients in two Dutch hospitals. Furthermore, a validated questionnaire was used to distinguish different PN grades.

**Results:**

Of the 120 patients included between January 2017 and August 2018, 84% had an inadequate vitamin D level (median vitamin D level 49.5 nmol/L [IQR 34–65 nmol/L]; mean age: 68 years [SD ± 7.7]; males: 58%). PN was reported by 69% of patients (n = 83); however, of these 83 patients, PN was not documented in the medical records of 52%. An association was found between lower vitamin D levels and higher incidence of PN in the total population (P = 0.035), and in the active MM patients (P = 0.016).

**Conclusion:**

This multi-centre cohort study showed that PN and hypovitaminosis D are common in MM patients, and addressing low vitamin D levels in the treatment of MM patients might be beneficial in reducing the risk of PN. More attention for PN is warranted, as PN is underreported by clinicians. Further research is needed to fully understand the implications of vitamin D in the development of PN in patients with MM.

**Clinical trial registration:**

Netherland Trial Register NL5835, date of registration July 28, 2016

**Supplementary Information:**

The online version contains supplementary material available at 10.1007/s00520-021-06414-3.

## Introduction

New strategies in the treatment of multiple myeloma (MM) greatly improved overall survival (OS) [1]. Despite the advances in treatment, patients frequently experience adverse events such as chemotherapy-induced peripheral neuropathy. Drugs that often induce peripheral neuropathy (PN) are the proteasome inhibitor bortezomib, and the immunomodulatory agents such as thalidomide [2–6]. These drugs are frequently given in sequence to MM patients. The cumulative dose is an important predictor for the occurrence of bortezomib-induced PN [7, 8]. Grade 2–3 neuropathy has been reported by 65% of patients using bortezomib when the ‘Indication for Common Toxicity Criteria (CTC) Grading of Peripheral Neuropathy Questionnaire’ (ICPNQ) was used [9], and other studies found percentages up to 70% of patients for these PN-inducing drugs [6, 10–12]. Such adverse events may make it necessary to adjust doses, or delay or terminate treatment, negatively influencing time to progression and survival [8, 11]. 

PN is not only induced by treatment, as studies have found percentages of PN in newly diagnosed MM patients in 13% [2], 11% [11], 54% [13], and 7.2% [14]. Also smouldering MM patients, who generally do not receive anti-myeloma treatment, may develop PN as a result of paraproteinemia [15, 16]. MM itself has the capacity to provoke PN via several mechanisms; the M protein produced by the malignant plasma cells, deposits of the light-chain immunoglobulins (amyloidosis) in nerve cells, or radicular or medullar compression in myeloma bone disease can directly damage nerve cells. Also, hyperviscosity of the blood as a result of the high M protein level slows down blood flow, causing neurological symptoms [17].

This high prevalence of PN makes it imperative to adjust doses or discontinue treatment with PN-inducing agents, use medication to alleviate the pain, or find interventions to prevent PN. One possible approach to prevent PN could be the administration of vitamin D. Vitamin D is a steroid hormone responsible for the regulation of calcium and phosphorus homeostasis. Once in the circulation, vitamin D is converted to 25-hydroxyvitamin D in the liver, and metabolized to the active metabolite 1,25-dihydroxyvitamin D in the kidneys [18]. 

Vitamin D has been shown to play an essential role in many conditions, such as bone mineralization [19], the inhibition of cancer cell proliferation [20], and insulin resistance [21]. Furthermore, vitamin D deficiency was found to be a predictor for poor overall survival in white, but not African American MM patients [22]. In relation to PN, several studies in non-insulin-dependent diabetes mellitus patients showed that an association exists between vitamin D deficiency and diabetic PN [23–26]. Also, in patients with breast cancer who were treated with paclitaxel, levels of vitamin D were significantly lower in patients with paclitaxel-induced PN compared to patients without PN [27]. 

A possible mechanism of vitamin D deficiency and PN could be the depletion of nerve growth factor [28]. In cancer patients who developed PN during treatment with bortezomib, thalidomide, or vincristine, depletion of this nerve growth factor occurred [29]. Other neuroprotective mechanisms of vitamin D include the ability to lower intracellular calcium levels [30]; the protection of the nerve cells against damage induced by reactive oxygen species [31]; or decreasing inflammatory reactions [32]. These observations suggest that vitamin D may have a role in the prevention of PN. Recently, two studies investigated this hypothesis in MM patients by measuring vitamin D levels and the severity and occurrence of PN [33, 34]. Both studies observed that vitamin D deficient patients were more prone to suffer from PN, but the studies lacked the use of a validated questionnaire specifically designed to measure PN in patients diagnosed with MM, as was used in this study. Patient-reported outcomes have demonstrated to be a valid, reliable, feasible, and precise approach to gather toxicities, and in clinical trials, they are the standard for subjective outcomes such as pain. Also, patient-reported toxicity assessments outperform clinician-reported ones, as a result of underreporting or insufficient questioning by clinicians [35, 36]. The studies excluded patients with smouldering MM, while these patients are also at risk of developing PN as previously described in this introduction. Another difference is the exposure to sunlight, as the Netherlands has a less sunny climate compared to the environments in which the other studies were conducted.

Therefore, the primary objective of this study was to determine the association between the vitamin D serum levels and PN in patients with smouldering or active MM, using a validated questionnaire. Secondary objectives were to gain insight into the percentage of patients with inadequate vitamin D serum levels and the prevalence of PN in a real-world MM population, and to determine to what extent the recording of PN in the patients’ records corresponds with the ICPNQ.

## Methods

### Study population

We approached all patients with smouldering or active MM, regardless of stage or previous treatment, who were under the supervision of a haematologist in the Medical Centre Leeuwarden (MCL) or Deventer Hospital (DH) in the Netherlands. Patients were at least 18 years old and were able to give informed consent. In concordance with the Medical Research Involving Human Subjects Act, approval of this study was obtained from the medical ethics committee. All patients provided written informed consent prior to participation. The study is registered in the Netherlands Trial Register (NL5835).

### Study design

In this multi-centre cohort study, blood samples were collected to determine vitamin D levels. Based on the Endocrine Society guideline and Summary of Product Characteristics of vitamin D [37, 38], patients were divided in four vitamin D categories; vitamin D insufficiency was defined as a vitamin D level below 75 nmol/L (= 30 ng/mL), vitamin D deficiency as a vitamin D level below 50 nmol/L (= 20 ng/mL), and patients were seriously deficient when vitamin D levels reached below 25 nmol/L (= 10 ng/mL). Due to its long half-life, 25-hydroxyvitamin D is considered a good indicator for vitamin D level, and we therefore determined 25-hydroxyvitamin D serum levels [39]. The blood samples were collected between January and April 2017 (MCL), and between January and August 2018 (DH). All blood samples were analyzed in the laboratory of the MCL by an immunochemical assay.

To measure the presence of PN, we used the ICPNQ, a validated questionnaire to distinguish different PN grades (grades 0 until 3) in MM patients [9]. The severity of the different items on the ICPNQ was assessed by using the Visual Analog Scale (VAS). Questionnaires were completed by one of the researchers in consultation with the patients before their clinical appointment with the haematologist. To determine the influence of treatment on the occurrence of PN, we calculated the cumulative dose for each drug the patient received since diagnosis until the questionnaire was completed. This was based on the dose per cycle as prescribed by the treating haematologist. Cransac et al. [40] showed that MM patients are highly adherent to their oral anti-myeloma drugs (94% according to the medication possession ratio, and 76% according to a questionnaire). A study in our hospital on patient adherence showed that patient adherence to oral anti-myeloma drugs was 100%, based on refill rates from the pharmacy (unpublished data). Therefore, the calculation of the cumulative oral dose was based on the dose as prescribed by the haematologist, in which 100% adherence was assumed. For the intravenously administered anti-myeloma agent bortezomib, the cumulative dose was calculated based on the dose as prepared in the hospital pharmacy. The use of oral vitamin D supplements was based on the information provided by the participants during the structured interview in which the use of vitamin D was explicitly questioned. We also compared the outcomes of our questionnaire with the information available in the patients’ electronic health records. We used the research data management program *Research Manager* to collect our data.

### Statistical analysis

Descriptive statistics were used for the baseline characteristics. The Kruskal–Wallis test was applied to analyze differences in medians. The exact chi-square test was used to analyze differences in the occurrence of PN in patients with different vitamin D levels. A *P* value < 0.05 indicated statistical significance. All analyses were performed using IBM SPSS Statistics 24.

## Results

### Baseline characteristics

During the study period, 120 patients were included. Table 1 presents the baseline characteristics of the total study population and the four vitamin D categories, separately. The mean age of the total population was 68 years (SD ± 7.7) and 58% was male. The majority of the patients was diagnosed with active MM (88%), and the median time since diagnosis was 35 months. Almost half of the patients used vitamin D supplementation prescribed by the general practitioner or purchased by themselves; patients with vitamin D supplementation had significantly higher vitamin D levels.

**Table 1:** Baseline characteristics of the total study population, and divided in the vitamin D subgroups. Mean and standard deviation (SD), median and interquartile range (IQR), or number and percentages (*n* (%)) are shown

Characteristic		Vitamin D				
	Total population (*n* = 120)	Seriously deficient (*n* = 17)	Deficient (*n* = 43)	Insufficient (*n* = 41)	Adequate (*n* = 19)	*P* value
Age, years; mean (SD)	68 (7.7)	70 (8)	67 (8)	68 (8)	69 (8)	0.56
Gender, male; *n* (%)	69 (58)	11 (65)	24 (56)	24 (58)	10 (53)	0.89
Race, Caucasian; *n* (%)	119 (99)	17 (100)	42 (98)	41 (100)	19 (100)	0.61
Active MM; *n* (%)	105 (88)	15 (88)	38 (88)	36 (88)	16 (84)	0.97
ISS stage						0.059
ISS I	20 (17)	4 (24)	6 (14)	10 (24)	0 (0)	
ISS II	32 (27)	3 (18)	12 (28)	16 (39)	1 (5)	
ISS III	29 (24)	5 (29)	11 (26)	7 (17)	6 (32)	
Unknown	39 (32)	5 (29)	14 (33)	8 (20)	12 (63)	
Plasma cells at diagnosis; median % (IQR)	24 (15–50)	30 (14–75)	20 (12–31)	30 (16–59)	20 (15–55)	0.26
Unknown; *n* (%)	14 (12)	3 (18)	5 (12)	4 (10)	2 (11)	
Time since diagnosis; median in months (IQR)	35 (16–71)	26 (17–41)	39 (12–70)	40 (24–74)	20 (9–31)	0.29
Diabetes; *n* (%)	15 (13)	4 (23)	3 (7)	6 (15)	2 (10)	0.34
Alcohol; *n* (%)						0.40
None	64 (53)	12 (70)	18 (42)	22 (54)	12 (63)	
1–3 per week	20 (17)	2 (12)	10 (23)	4 (10)	4 (21)	
4–9 per week	24 (20)	1 (6)	11 (26)	10 (24)	2 (11)	
≥ 10 per week	12 (10)	2 (12)	4 (9)	5 (12)	1 (5)	
Vitamin D supplementation; *n* (%)	54 (45)	2 (12)	11 (26)	26 (63)	15 (79)	< 0.001

Vitamin D categories: seriously deficient =  < 25 nmol/L; deficient = 25–50 nmol/L; insufficient = 50–75 nmol/L; adequate ≥ 75 nmol/L

*MM*, multiple myeloma; *ISS*, international staging system

### Vitamin D and peripheral neuropathy

The median vitamin D level for the total population was 50 nmol/L (interquartile range [IQR] 34–65 nmol/L), and for the seriously deficient, deficient, insufficient, and adequate vitamin D subgroups 21, 38, 59, and 87 nmol/L, respectively. The majority of the patients (84%) had a vitamin D level < 75 nmol/L.

**Fig. 1:**
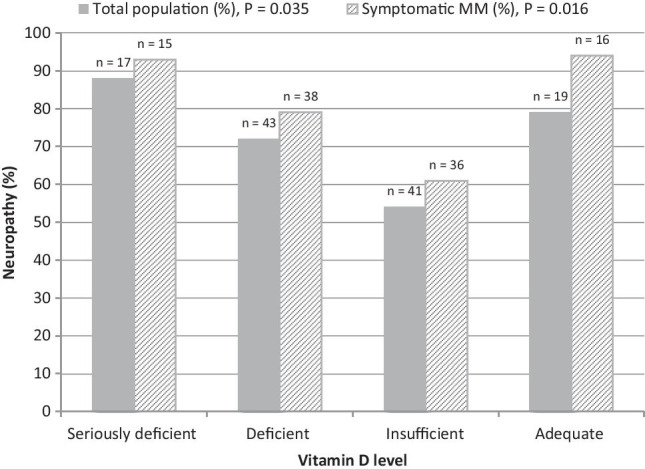
Percentage of patients with peripheral neuropathy in the four vitamin D subgroups for the total population (solid bars) and the active MM patients (dashed bars)

Overall, the ICPNQ resulted in 83 out of 120 patients (69%) with any grade of PN. Of these, 26, 38, and 5% experienced PN grade 1, 2, or 3, respectively. Two out of the 15 patients (13%) with smouldering MM experienced PN; the vitamin D status of these two patients was seriously deficient and deficient. Figure 1 shows the percentage of patients with PN in the vitamin D subgroups for the total population and the active MM patients. With increasing vitamin D levels and decreasing PN, vitamin D levels are associated with the occurrence of PN (total MM population: *P* = 0.035; active MM patients: *P* = 0.016). However, with 79% and 94% PN (total population and active patients, respectively), this was not the case for the adequate vitamin D subgroup.

The symptoms of PN that were most often reported were numbness, a tingling sensation, pain, changes in the perception of temperature, and loss of muscle strength (Supplementary Table 1). The symptoms with the highest VAS scores were changes in the sensation of temperature, and pain in the fingers, toes, feet, and lower legs (Supplementary Table 2).

PN was reported by 69% of patients (n = 83); however, of these 83 patients, PN was not documented in the medical records of 52%, indicating underreporting.

### Cumulative dose and peripheral neuropathy

Table 2 provides an overview of the intensity of treatment that patients received in the vitamin D subgroups, presented as cumulative doses per month. The time since diagnosis (see Table 1), and cumulative doses for the deficient and insufficient vitamin D groups were similar, as well as for the seriously deficient and adequate vitamin D groups, but higher cumulative doses of neurotoxic drugs per month were found for the adequate vitamin D group.

**Table 2:** Median and interquartile range (IQR) for the intensity of treatment with bortezomib, thalidomide or lenalidomide per month for each vitamin D subgroup

	Vitamin D level											
	Seriously deficient (n = 17)			Deficient (n = 43)			Insufficient (n = 41)			Adequate (n = 19)		
Bortezomib;mg/m^2^ per month	1.1	(0.63 – 1.4)	n = 12	0.88	(0.26 – 1.7)	n = 35	0.92	(0.63 – 1.9)	n = 33	1.8	(0.56 – 3.2)	n = 14
Thalidomide;mg per month	511	(187 – 615)	n = 6	298	(72 – 2783)	n = 6	555	(118 - 1438)	n = 14	834	(291 – 1450)	n = 4
Lenalidomide;mg per month	81	(45 – 160)	n = 8	76	(25 – 137)	n = 11	60	(31 – 137)	n = 15	88	(30 – 98)	n = 5

## Discussion

Our cohort study illustrates that PN is a major problem in patients with MM. More than two-thirds of the patients experienced any form of PN, with PN grade 2 as the most reported. Using a validated questionnaire, we showed that there is an association between low vitamin D levels and the occurrence of PN. Inadequate vitamin D levels were measured in 84% of the patients when using the international standard for deficiencies (< 75 nmol/L). Furthermore, PN was measured more often compared to the observations described in the health records by the treating haematologists, suggesting that PN is severely underreported in daily clinical practice.

The association between vitamin D and PN found in this study suggests a neuroprotective effect of vitamin D. In contrast, this protective effect was not observed in MM patients with the highest vitamin D levels. A possible explanation may be found in the shorter duration of disease, and the more intense treatment with higher cumulative doses of neurotoxic drugs than the patients in the other vitamin D subgroups, as shown in Tables 1 and 2. This group of patients actually presented with a comparable occurrence of PN as in the seriously deficient vitamin D group, also relatively early in their treatment phase, but with a less intense treatment regimen. Furthermore, the subgroup analyses with active MM patients showed an even more pronounced effect, with PN in 94% of the patients with adequate vitamin D levels. This supports the hypothesis that the higher incidence in the adequate vitamin D group is due to anti-myeloma therapy, and suggests that the adequate vitamin D levels offer insufficient nerve protection in these heavily treated patients. It therefore seems that the patients with adequate vitamin D levels had a different risk profile with regard to the occurrence of PN.

This study also showed that, as expected, the incidence of PN is lower in smouldering MM patients, but nevertheless PN does occur in these patients. Both smouldering patients with PN in our study had deficient vitamin D levels, did not receive anti-myeloma treatment, and did not have any known PN-inducing comorbidities. Exposure to adequate vitamin D levels might prevent PN, especially since these patients are generally not treated with neurotoxic anti-myeloma therapy.

Assessing vitamin D levels is no standard of care in MM patients in the Netherlands. Nevertheless, Dutch guidelines do recommend supplementation of vitamin D in the elderly [41]. When we consider that the MM population consists of elderly patients, and almost 40% of the patients with an inadequate vitamin D level received vitamin D supplementation in our study, the high proportion of MM patients with insufficient vitamin D levels was remarkable. Therefore, if supplementation is initiated, monitoring of vitamin D levels should be mandatory.

Our results complement previous findings regarding the high prevalence of vitamin D deficiency, and the relationship between vitamin D and PN in MM patients. Wang et al. included 111 patients, of whom 42% were vitamin D deficient (16%) or insufficient (26%). The authors did not find a relationship between deficient or insufficient vitamin D levels and the occurrence of PN, but they did find that patients with insufficient vitamin D levels had worse PN (> grade 2). Recently, a small study in Australia with 41 patients investigated the prevalence of vitamin D deficiency, and examined potential associations with myeloma severity. Despite the sunny climate, only 39% of the patients had sufficient (> 72 nmol/L) vitamin D levels. In addition, vitamin D deficient patients experienced more PN than the sufficient patients (73 vs. 33%, *P* = 0.03). In contrast with our study, these studies did not use a validated questionnaire designed to distinguish PN grades in MM patients, and they only included active patients on anti-myeloma therapy.

This study has some limitations. First, the study population mainly consisted of patients with a Caucasian background, making it difficult to extrapolate these results to patients with other ethnic backgrounds. Second, we designed a cross-sectional cohort study of MM patients and included patients at different disease stages, potentially introducing confounding. Different chemotherapy regimens and the duration of the disease could have influenced the results. In fact, we speculate that these differences in part obscured the efficacy of vitamin D in our adequate vitamin D subgroup. Also, the use of vitamin D supplementation at the start of the study might be a proxy for the earlier existence or worsening of PN, i.e. confounding by indication. As supplementation with vitamin D is currently not a recognized treatment for PN, and its supplementation is not actively advised by treating haematologists nor mentioned in current guidelines, we do not expect this to have affected the results. Strengths of our study are the use of a validated questionnaire designed to measure PN grades in MM patients, and the questionnaire was always filled in by one of the trained researchers. Furthermore, we included patients in two hospitals situated in different areas of the Netherlands, and all blood samples were analyzed in one laboratory.

The current study emphasizes the need for adequate reporting of PN, especially in the early stage of treatment where the occurrence of PN seems to be higher. The poor correspondence of the ICPNQ and PN in the electronic health records indicates the underreporting of PN in MM patients in clinical practice. Patient-reported outcomes are a feasible and reliable option that enables the detection of symptoms that are missed by the treating haematologists. Therefore, physicians should emphasize on patient-reported signs of PN, in order to intervene appropriately and prevent further nerve damage.

In conclusion, this study shows that both PN and hypovitaminosis D are common in MM patients, and addressing low vitamin D levels in the treatment of MM patients might be beneficial in reducing the risk of PN associated with neurotoxic drugs. Importantly, more attention for PN is warranted, as PN is underreported by clinicians. Further research on causality is needed to fully understand the implications of vitamin D in the development of PN, especially in the earlier stage of MM treatment.

## Supplementary Information

Below is the link to the electronic supplementary material.

Supplementary file1 (DOCX 22.9 KB)

## Data Availability

The data that support the findings of this study are available from the corresponding author upon reasonable request.

## Code availability

N/A.
