# Supplementary material for: High prevalence of peripheral neuropathy in multiple myeloma patients and the impact of vitamin D levels, a cross-sectional study
Source: Support Care Cancer. 2021 Jul 17;30(1):271–8. doi: 10.1007/s00520-021-06414-3 (PMC8636433; doi:10.1007/s00520-021-06414-3)
Supplement: Supplementary file 1 — Supplementary file1 (DOCX 22.9 KB) [file 520_2021_6414_MOESM1_ESM.docx]

Supplementary Table 1. Number of patients that responded “yes” to the questions of the ICPNQ.

|  | Vitamin D level | | | | | | | |  | |
| --- | --- | --- | --- | --- | --- | --- | --- | --- | --- | --- |
|  | Seriously deficient; n (%)  n = 17 | | Deficient; n (%)  n = 43 | | Insufficient; n (%)  n = 41 | | Adequate; n (%)  n = 19 | | Total population; n (%)  n = 120 | |
| **Sensory symptoms: do you experience a change in feeling?** | | | | | | | | |  | |
| When touched | 2 | (11.8) | 4 | (9.3) | 7 | (17.1) | 6 | (31.6) | 19 | (15.8) |
| Numbness | 13 | (76.5) | 24 | (55.8) | 18 | (43.9) | 10 | (52.6) | 65 | (54.2) |
| Tingling | 8 | (47.1) | 17 | (39.5) | 14 | (34.1) | 12 | (63.2) | 51 | (42.5) |
| Changes in temperature sensation | 6 | (35.3) | 16 | (37.2) | 4 | (9.8) | 9 | (47.4) | 35 | (29.2) |
| Pain (burning, stabbing, stinging or cramping) | 10 | (58.8) | 16 | (37.2) | 12 | (29.3) | 9 | (47.4) | 47 | (39.2) |
| The pain is continuous | 1 | (5.9) | 3 | (7.0) | 4 | (9.8) | 2 | (10.5) | 10 | (8.3) |
| The pain is intermittent | 9 | (52.9) | 12 | (27.9) | 8 | (19.5) | 7 | (36.8) | 36 | (30.0) |
| **Autonomic symptoms: are you experiencing changes in the following function?** | | | | | | | | |  | |
| Frequent urination | 4 | (23.5) | 11 | (25.6) | 2 | (4.9) | 4 | (21.1) | 21 | (17.5) |
| Difficulty holding urination or fully emptying bladder | 1 | (5.9) | 2 | (4.7) | 1 | (2.4) | 3 | (15.8) | 7 | (5.8) |
| Sexual intercourse | 5 | (29.4) | 10 | (23.3) | 5 | (12.2) | 6 | (31.6) | 26 | (21.7) |
| Constipation | 3 | (17.6) | 1 | (2.3) | 3 | (7.3) | 2 | (10.5) | 9 | (7.5) |
| Diarrhoea | 4 | (23.5) | 3 | (7.0) | 3 | (7.3) | 3 | (15.8) | 13 | (10.8) |
| Dizziness when standing up | 3 | (17.6) | 11 | (25.6) | 5 | (12.2) | 2 | (10.5) | 21 | (17.5) |
| Heart palpitations | 0 | (0.0) | 4 | (9.3) | 2 | (4.9) | 3 | (15.8) | 9 | (7.5) |
| Increased sweating | 2 | (11.8) | 8 | (18.6) | 3 | (7.3) | 5 | (26.3) | 18 | (15.0) |
| Reduced sweating | 3 | (17.6) | 1 | (2.3) | 1 | (2.4) | 2 | (10.5) | 7 | (5.8) |
| **Motoric symptoms: are you experiencing a loss of muscle strength?** | | | | | | | | |  | |
| Muscle strenght | 6 | (35.3) | 14 | (32.6) | 9 | (22.0) | 10 | (52.6) | 39 | (32.5) |
| **Motoric symptoms: are you experiencing any problems carrying out the activities below?** | | | | | | | | |  | |
| Dressing/undressing without assistance | 2 | (11.8) | 0 | (0.0) | 1 | (2.4) | 0 | (0.0) | 3 | (2.5) |
| Washing unaided, washing and combing hair unaided | 2 | (11.8) | 0 | (0.0) | 1 | (2.4) | 0 | (0.0) | 3 | (2.5) |
| Holding cutlery and eating unaided | 0 | (0.0) | 0 | (0.0) | 0 | (0.0) | 1 | (5.3) | 1 | (0.8) |
| Walking independently | 2 | (11.8) | 1 | (2.3) | 1 | (2.4) | 1 | (5.3) | 5 | (4.2) |
| Opening/closing doors unaided | 0 | (0.0) | 0 | (0.0) | 0 | (0.0) | 0 | (0.0) | 0 | (0.0) |
| Driving a car unaided | 1 | (5.9) | 1 | (2.3) | 1 | (2.4) | 1 | (5.3) | 4 | (3.3) |
| Using the telephone/computer keyboard | 1 | (5.9) | 3 | (7.0) | 2 | (4.9) | 2 | (10.5) | 8 | (6.7) |
| (Un)buttoning a blouse | 2 | (11.8) | 4 | (9.3) | 7 | (17.1) | 4 | (21.1) | 17 | (14.2) |
| Tie shoelaces | 2 | (11.8) | 1 | (2.3) | 1 | (2.4) | 1 | (5.3) | 5 | (4.2) |
| Writing | 1 | (5.9) | 3 | (7.0) | 3 | (7.3) | 2 | (10.5) | 9 | (7.5) |
| Preparing a meal | 0 | (0.0) | 0 | (0.0) | 1 | (2.4) | 0 | (0.0) | 1 | (0.8) |

Supplementary Table 2. Intensity of the sensory neuropathy symptoms.

|  | VAS scores^#^ for intensity by vitamin D level | | | | | | | |  | |
| --- | --- | --- | --- | --- | --- | --- | --- | --- | --- | --- |
|  | Seriously deficient;  mean (SD)  n = 17 | | Deficient;  mean (SD)  n = 43 | | Insufficient;  mean (SD)  n = 41 | | Adequate;  mean (SD)  n = 19 | | Total population;  mean (SD)  n = 120 | |
| **Sensory symptoms: do you experience a change in feeling?** | | | | | | | | |  | |
| When touched | 4.3 | (2.8) | 5.0 | (0.8) | 3.5 | (1.1) | 3.1 | (1.4) | 3.7 | (0.6) |
| Numbness | 4.3 | (0.8) | 4.3 | (0.5) | 3.9 | (0.5) | 4.6 | (0.8) | 4.2 | (0.3) |
| Tingling | 4.5 | (0.9) | 4.2 | (0.6) | 4.2 | (0.5) | 4.1 | (0.7) | 4.2 | (0.3) |
| Changes in temperature sensation | 4.8 | (1.6) | 4.8 | (0.6) | 6.6 | (1.7) | 4.2 | (1.0) | 4.8 | (0.5) |
| Pain (burning, stabbing, stinging or cramping) | 6.5 | (1.1) | 6.4 | (0.5) | 6.0 | (0.8) | 4.4 | (1.0) | 5.9 | (0.4) |

^#^ Mean score (range 0 – 10) for the sum of places (toes, feet, lower legs, fingers, hands, lower forearms) where change in feeling is experienced. VAS: visual analog scale.
